# The Non-Transmission of Syphilis by Vaccination

**Published:** 1865-10

**Authors:** 


### The Non-Transmission of Syphilis by Vaccination.

This is the title of a peper by Professor W. Boeck of Christiana, a translation of which was read at the late meeting of the British Medical Association. The following outline of it we find in the *British Medical Journal:*

"In it the author stated that he had most carefully examined the question, whether syphilis could be transmitted by vaccination; and had been unable to find any evidence in favor of such transmission either in published records or from experiments performed by himself. He related instances in which he had vaccinated syphilitic children, and had endeavored, but without producing any result, to inoculate with the matter obtained from them two patients suffering from elephantiasis. He considered that no doubt should be thrown on vaccination unless on the most convincing evidence; and, while he did not deny that syphilis might be transmitted by vaccine matter, he must withhold his belief that such an event could occur until he saw it. The facilities for observation were great in Norway; but the transmission of syphilis by vaccination had never there been observed."

